# Neurological abnormalities and neurocognitive functions in healthy elder people: A structural equation modeling analysis

**DOI:** 10.1186/1744-9081-7-32

**Published:** 2011-08-10

**Authors:** Raymond CK Chan, Ting Xu, Hui-jie Li, Qing Zhao, Han-hui Liu, Yi Wang, Chao Yan, Xiao-yan Cao, Yu-na Wang, Yan-fang Shi, Paola Dazzan

**Affiliations:** 1 Neuropsychology and Applied Cognitive Neuroscience Laboratory, Institute of Psychology, Chinese Academy of Sciences, Beijing, China; 2 Key Laboratory of Mental Health, Institute of Psychology, Chinese Academy of Sciences, Beijing, China; 3 Graduate School, Chinese Academy of Sciences, Beijing, China; 4 School of Psychology, Beijing Normal University, Beijing, China; 5 Institute of Psychiatry, King's College, London, UK

**Keywords:** neurological soft signs, neurocognitive impairments, elderly, Chinese

## Abstract

**Background/Aims:**

Neurological abnormalities have been reported in normal aging population. However, most of them were limited to extrapyramidal signs and soft signs such as motor coordination and sensory integration have received much less attention. Very little is known about the relationship between neurological soft signs and neurocognitive function in healthy elder people. The current study aimed to examine the underlying relationships between neurological soft signs and neurocognition in a group of healthy elderly.

**Methods:**

One hundred and eighty healthy elderly participated in the current study. Neurological soft signs were evaluated with the subscales of Cambridge Neurological Inventory. A set of neurocognitive tests was also administered to all the participants. Structural equation modeling was adopted to examine the underlying relationship between neurological soft signs and neurocognition.

**Results:**

No significant differences were found between the male and female elder people in neurocognitive function performances and neurological soft signs. The model fitted well in the elderly and indicated the moderate associations between neurological soft signs and neurocognition, specifically verbal memory, visual memory and working memory.

**Conclusions:**

The neurological soft signs are more or less statistically equivalent to capture the similar information done by conventional neurocognitive function tests in the elderly. The implication of these findings may serve as a potential neurological marker for the early detection of pathological aging diseases or related mental status such as mild cognitive impairment and Alzheimer's disease.

## Background

Neurological soft signs are minor neurological abnormalities in sensory and motor function commonly reported in disorders such as schizophrenia spectrum disorders [1,2], autism spectrum disorders [3], and obsessive-compulsive disorders [4]. Recent studies suggest that healthy people at different developmental stages in their lives also exhibit differential base-rates of neurological signs [5-7]. This is particularly true for elderly individuals, because neurological signs increase with advancing age [8]. Past research on neurological abnormalities in elderly individuals has primarily focused on evaluating extrapyramidal disturbances or focal signs, which are collectively known as "hard" signs [9-13]. These are impairments of basic motor and sensory function, corresponding to alterations in focal and localized brain areas, such as the basal ganglia [14], and they have been reported as increased in aging populations. In contrast, although minor neurological abnormalities have been observed in pathological aging populations, such as Alzheimer's disease and Parkinson's disease, the prevalence of neurological soft signs (such as rapid alternate finger tapping or astereognosis) in the healthy aging population has not been examined.

In contrast to hard signs, the presence of neurological soft signs has been thought to indicate a diffuse damage of the brain, not related to damage of a specific brain region. However, recent structural imaging studies show that neurological soft signs are significantly associated with volume changes in specific brain regions, and reductions in gray matter in both neuropsychiatric disorders and healthy volunteers [15,16]. Moreover, most recent functional imaging studies also show that, although no specific single region is responsible for these neurological soft signs, there is a specific neural network linking the right inferior prefrontal cortex to the middle prefrontal cortex underlying their presence [17,18].

This is particularly interesting considering that the frontal hypothesis of cognitive aging suggests that the prefrontal cortex deteriorates earlier and disproportionately in comparison to the rest of the cortex [19], showing a decline in volume, white matter density, and synaptic density [20]. Consistent with changes in frontal cortex, is the robust finding of a general decline in executive function (critically regulated by the frontal cortex) associated with physiological aging processes (e.g., [21-23]). It is of note that in fact neurological soft signs and cognitive deficits have been thought to be functional correlates of the same pathophysiological substrate.

Interestingly, a recent study from our group [24], adopting structural equation modeling, showed that motor coordination, sensory integration, and disinhibition contributed to the latent construct of neurological soft signs, whereas the subset of neurocognitive function tests contributed to the latent constructs of executive attention, verbal memory, and visual memory. Greater evidence of neurological soft signs is associated with more severe impairment of executive attention and memory functions in a group of patients with schizophrenia and healthy controls independently.

Therefore, it is important to explore the prevalence rates of neurological soft signs, and their relationship to executive and other neurocognitive functions in the cognitively intact elderly people. Unfortunately, existing studies have been limited to the evaluation of neurological hard signs, and almost all of them have evaluated individuals of Caucasian origin. In contrast, very little is known about individuals from other ethnic groups such as the Chinese.

The purpose of the current study was to examine the underlying relationship between neurological soft signs and neurocognitive function in a sample of healthy Chinese elderly people by means of a standardized neurological signs scale. Given the previous findings of more or less equivalent constructs between neurological soft signs and neurocognitive functions in schizophrenia and healthy young adults, it was hypothesized that a similar structural equation model would be observed in healthy elderly participants

## Method

### Ethics Statement

The present study was approved by the ethics committee of the Institute of Psychology, Chinese Academy of Sciences. Written informed consent was obtained by participants.

### Participants

One hundred and eighty healthy elderly participants (86 men and 94 women) were recruited from several provinces of China, through household visits and local advertisements. Exclusion criteria were: (1) a history of head trauma resulting in loss of consciousness for > 1 h; (2) a diseases of the central nervous system; (3) a history of any psychotic disorder; (4) current and past alcohol or substance abuse.

All participants were evaluated with the Mini Mental State Examination (MMSE) [25], which includes tests of learning, memory, and verbal ability, to assess cognitive dysfunction. An MMSE score lower than 24 points was considered to indicate a probable diagnosis of Alzheimer Disease [26].

### Materials and procedure

The Cambridge Neurological Inventory (CNI) was used for the assessment of neurological soft signs [27]. The CNI has been applied to different age groups in both healthy and clinical Chinese populations [6,28,29]. The administration the CNI has been described elsewhere (e.g., [6,27]). In brief, The CNI soft signs assessment consisted of motor coordination (e.g., rapid finger tapping, fist-edge-palm), sensory integration (e.g., astereognosis, agraphesthesia), and inhibition (e.g., mirror movement, eye blink while performing eye tracking). Each item were assessed as either "absent" (which covered the normal or equivocal scale scores) or "present" (which covered the abnormal or grossly abnormal scale scores). Items that could be scored on both the left and the right were each treated as independent scores. All items were rated by a trained research assistant. We administered the CNI in a standardized manner, as specified for each item, and according to a fixed order.

The logical memory and visual reproduction subtests selected from the Chinese version of the Wechsler Memory Scale-Revised [30] were used to measure verbal memory and visual memory. For logical memory, the participants was told a short story and asked to recall it immediately and again 30 minutes later. For visual reproduction, the participant was shown two pictures (one at a time) for 10s each, and asked to draw them immediately and again 30 min later.

Working memory was assessed by the Chinese version of the Letter-Number Span Test (Chinese LN span) [31] and the forward and backward digit span tests. In the Letter-Number sequencing task, the experimenters verbally presented a specified sequence of random digits (1 to 9) mixed with Chinese characters (in a specific sequence, such as A, B, C in English) at a rate of one per second. Participants were asked to rearrange the order so that the digits came first, from small to big, and then the characters, in sequence. The number of digits and letters increased with 1 digit or character until the participant incorrectly repeated two trials of the same length.

### Data analysis

We focused on evaluating the relationship between neurological soft signs and neurocognitive function with structure equation model (SEM). It is hypothesized that neurocognitive functions co-vary with neurological soft signs. Neurocognitive functions and neurological soft signs were latent variables, as measured by the corresponding scales. Specifically, Logical Memory Immediate Recall and Logical Memory Delayed Recall were indices of "Verbal Memory"; and the Visual Reproduction Immediate Recall and Visual Reproduction Delayed Recall were indices of "Visual Memory"; the number of correct passed and the number of longest passed items described the "Working Memory". On the other hand, the Motor Coordination, Sensory Integration, and Disinhibition contributed to "Neurological Soft Signs". The relationship between neurocognitive functions, i.e., Verbal Memory, Visual Memory, and Working Memory and neurological soft signs (Motor Coordination, Sensory Integration, and Disinhibition) were covariant.

The SEM was conducted using LISREL 8.70 for Windows. All the variables were standardized before being entered in the model. Five fit indices were recorded to evaluate the fitness of goodness of the model. They are the Root Mean Square Error of Approximation (RMSEA), Normed Fit Index (NFI), Non-Normed Fit Index (NNFI), Comparative Fit Index (CFI), and Goodness of Fit Index (GFI). These indices represent the improvement in fit between the assumed model and the baseline model of uncorrelatedness between the observed variables. The first four fit indices values of .90 or above, and an RMSEA value of .08 or less indicated the model adequately fits the data [32].

## Results

The age of the participants ranged between 60 to 96 years (mean age 69.35, SD = 6.53). We divided 180 participants into three group according to the age. The summary scores of neurocognitive function and soft signs were displayed in Table 1. There was a significant tendency for groups with elder subjects have worse performance on logical memory, visual memory (delay score) and soft signs total and sensory integration score. However, the tendency was plus to nonsignificant and significant for the neurocognitive tasks and soft signs, respectively, when education was took in to account as covariance in ANCOVA.

**Table 1:** Descriptive statistics for neurocognitive functions and neurological soft signs scores for different age groups

Age range	60-69 (n = 109)		70-79 (n = 56)		> = 80 (n = 15)		ANOVA (*df *= 2,177)	
	
	Mean	SD	Mean	SD	Mean	SD	F	p-value
Age (years)	65.07	2.80	73.91	2.79	83.40	4.12	358.28	0.000
Education (number of years)	26.37	3.02	25.20	3.34	24.53	3.64	3.92	0.022
MMSE	7.70	3.90	5.57	4.52	5.27	5.70	5.73	0.004
Logical Memory	8.63	3.89	6.93	3.94	6.27	3.79	4.98	**0.008**
Logical Memory (Delay)	6.56	3.74	4.70	3.54	4.80	3.38	5.48	**0.005**
Visual reproduction	17.06	5.21	15.25	6.16	14.47	5.33	2.85	0.061
Visual reproduction (Delayed)	14.94	6.20	12.14	6.78	11.73	5.15	4.53	**0.012**
LN span items passed	8.28	4.47	6.96	4.14	7.52	6.96	1.55	0.216
LN span longest passed	3.83	1.80	3.36	1.84	3.11	2.51	1.76	0.175
Motor coordination	1.80	1.63	2.25	1.80	2.40	1.45	1.85	0.160
Sensory integration	1.70	1.44	2.57	1.54	2.80	1.61	8.46	**0.000**
Disinhibition	1.73	1.40	2.04	1.40	2.07	1.39	1.05	0.353
Total NSS score	5.23	3.21	6.86	3.39	7.27	3.31	5.99	**0.003**

LN span: Letter-Number Span Test; MMSE: Mini Mental Status Examination; NSS: Neurological Soft Signs

There were no differences in age between males and females (t = 0.48, p = 0.63). The mean years of education was 6.84 (SD = 4.38), and education in males was higher than in females (t = 2.07, p = 0.04). The MMSE scores did not differ between males and females (t = 1.15, 0.25), with a mean of 25.85 (SD = 3.23). Table 2 displays the means, SDs and the independent sample t-test results comparison across gender. In neurocognitive and neurological soft signs performance there were also no significant differences between males and females. There was however .05 <*p *< .10 in Logical Memory performance, where males performed better than females, and for sensory integration, where females tended to have more sensory integration signs than males. Table 3 shows prevalence rate, chi-square value, and Fisher's p of neurological soft signs for each item. Only the rate of one item (Graphesthesia R) was significantly different between males and females. On average, there were no significant differences found between males and females in the prevalence rate of individual items of neurological soft signs.

**Table 2:** Comparison of neurocognitive functions and neurological soft signs between male and female

	Male (N = 86)		Female (N = 94)		t-test (df = 178)	
	
	Mean	SD	Mean	SD	T	p-value
Logical memory	8.50	4.06	7.36	3.85	1.93	0.055
Logical memory (Delayed)	6.31	3.87	5.39	3.58	1.66	0.099
Visual reproduction	16.85	4.96	15.76	6.08	1.33	0.187
Visual reproduction (Delayed)	14.08	6.11	13.55	6.77	0.55	0.582
LN span items passed	8.16	4.74	7.46	5.24	0.86	0.389
LN span longest passed	3.85	1.90	3.36	2.08	1.53	0.129
Motor coordination	2.00	1.71	1.98	1.66	0.08	0.933
Sensory integration	1.85	1.35	2.26	1.68	-1.79	0.075
Disinhibition	1.69	1.36	2.01	1.43	-1.56	0.120
Total NSS score	5.53	3.32	6.24	3.38	-1.42	0.158

LN span: Letter-Number Span Test; MMSE: Mini Mental Status Examination; NSS: Neurological Soft Signs

**Table 3:** Prevalence rates of individual neurological soft signs items

Item	Male (N = 86)		Female (N = 94)			
	**No**.	**%**	**No**.	**%**	**chi-square**	**Fisher's Exact p**

Finger-thumb tapping L	1	1.16%	2	2.13%	0.255	1.000
Finger-thumb tapping R	1	1.16%	0	0.00%	1.099	0.478
Finger-thumb opposition L	13	15.12%	21	22.34%	1.530	0.255
Finger-thumb opposition R	19	22.09%	19	20.21%	0.095	0.855
Mirror 1 L	14	16.28%	20	21.28%	0.732	0.448
Mirror 1 R	21	24.42%	19	20.21%	0.460	0.591
Diadochokinesia L	10	11.63%	8	8.51%	0.485	0.620
Diadochokinesia R	9	10.47%	4	4.26%	2.585	0.150
Mirror 2 L	8	9.30%	15	15.96%	1.785	0.264
Mirror 2 R	3	3.49%	10	10.64%	3.426	0.085
Fist-edge palm L	45	52.33%	46	48.94%	0.206	0.658
Fist-edge palm R	34	39.53%	41	43.62%	0.308	0.650
Oszeretsky test	40	46.51%	45	47.87%	0.033	0.882
Extinction	2	2.33%	5	5.32%	1.077	0.447
Finger Agnosia L	45	52.33%	54	57.45%	0.476	0.549
Finger Agnosia R	33	38.37%	43	45.74%	1.001	0.366
Stereognosia L	7	8.14%	4	4.26%	1.181	0.365
Stereognosia R	3	3.49%	6	6.38%	0.792	0.051
Graphesthesia L	18	20.93%	30	31.91%	2.771	0.129
Graphesthesia R	26	30.23%	43	45.74%	4.572	**0.046**
L-R Orientation	25	29.07%	27	28.72%	0.003	1.000
Saccade BLK	5	5.81%	9	9.57%	0.885	0.412
Saccade Head	36	41.86%	31	32.98%	1.516	0.280
Wink	30	34.88%	41	43.62%	1.434	0.285
Go/No-Go	28	32.56%	44	46.81%	3.800	0.067

**Figure 1:**
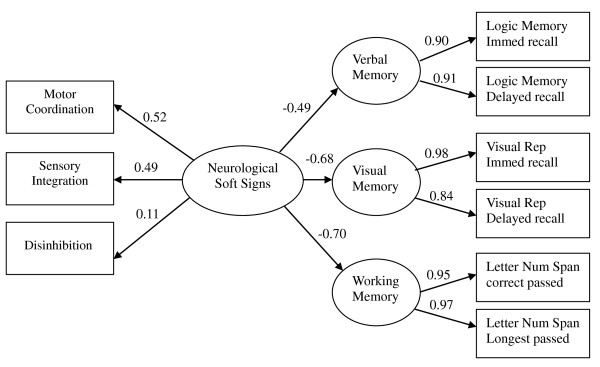
**The structure model of influence of soft signs on cognitive function in elderly**. Chi-square = 60.98, p < 0.001, df = 30, NFI = 0.93, NNFI = 0.96, CFI = 0.97, GFI = 0.93, RMSEA = 0.076, Standardized RMR = 0.064.

The model showed a good fit of the structure in this elderly sample. Figure 1 shows the structure path and the loadings of the model. The structural paths from neurological soft signs to verbal memory, visual memory and working memory were -0.49, -0.68, and -0.70, respectively. All loadings were statistically significant except the one from neurological soft signs and disinhibition. The chi-square was 60.98 (p < 0.001) with degree of freedom = 30. The fitness index indicated relatively well fit of the model, RMSEA = 0.076, NFI = 0.93, NNFI = 0.96, CFI = 0.97, GFI = 0.93, and Standardized RMR = 0.064.

## Discussion

The findings suggest that neurological soft signs are very common among elderly people. Like neurological hard signs such as basal ganglia signs, soft signs appear also to increase in prevalence with advancing age [5,10]. Very few data have been reported on the lifespan performance of neurological soft signs in healthy sample. A longitudinal study has found that neurological soft signs diminished with age from childhood to adolescence [33]. The current study did suggest that the prevalence of neurological soft signs increase with age for elderly people. Though there is formal publishable data specifically for the normal aging sample, the data implicated by the healthy volunteers as controls for Alzheimer's disease suggest a base-rate of soft signs in this sample, with motor coordination signs as the most commonly endorsed items [29]. The significant and robust associations found between neurological soft signs and neurocognitive function were also consistent with findings from studies conducted in individuals with schizophrenia and Alzheimer's disease [29,34-36] suggesting that maybe there is common neural substrates between neurological soft signs and neurocognitve function. Most recently, Chan et al. [24] adopted a structural equation modeling approach evaluating neurological soft signs and conventional neurocognitive functioning in schizophrenia and healthy volunteers, and showed that these two constructs (neurological soft signs and neurocognitive functioning) significantly overlap, with regression coefficients higher than 0.5, and capture a similar information of these two constructs. Interestingly, the same model was observed in the healthy controls. Taken together, these findings suggest that this model is relatively robust. However, it should be noted that the statistical model cannot confirm that neurological soft signs and neurocognitive function share the same neural substrates. The underlying neural mechanism needs to be further studied by rigorous experimental design for a better understanding of the relationship between neurological soft signs and neurocognitive functions in the near future.

The negative correlation between neurological soft signs and cognitive performance in our study is consistent with the previous findings that have shown that there were significant differences in neurological soft signs between older people with and without cognitive impairment in the Chinese setting. For example, Lam et al. [29] showed that patients with Alzheimer's disease have a significantly higher prevalence of neurological soft signs than those without dementia. In their clinical sample, Lam et al. [29] found the CNI soft signs subscales to be very sensitive in discriminating between cases with dementia and cases without, and this was particularly the case for motor coordination signs and sensory integration signs. Our findings further support this claim; at least it is sensitive enough to detect cognitively impaired and intact cases in terms of motor coordination. Genetic studies from schizophrenia also suggest that α 7 nicotinic cholinergic receptor [37], catechol-O-methytransferase (COMT) and GRM3 genetic variation [38] may be related to the presence of neurological soft signs. Taken together, the use of neurological soft signs may serve as potential sensitive bedside screening tool for neuropsychiatric or neurodegenerative disorders.

The current study has several limitations. First, our findings should be considered preliminary. Because of the small sample size and the non-stratified sample selection, they cannot be considered to represent prevalence rates of neurological soft signs in Chinese elderly people. The data should not be generalized to the elderly population of China, which has a total population of 1.3 billion. About 100.45 million people are over 65 years of age, occupying 7.69% of the overall population in China [39]. Secondly, we could not subdivide the sample into subgroups of old-old and oldest-old, because of the relatively limited number of people over the age of 85. Empirical findings suggest that this oldest-old age group is particularly at risk of developing neurological disorders or neurodegenerative illnesses such as dementia or Alzheimer's disease, with prevalence rates as high as 47% [40]. It could be expected that these age group would therefore show even higher neurological soft signs rates. Third, the current study did not assess IQ, which has been considered to be highly associated with neurological soft signs (e.g., [28]). However, since there were no differences in education (an indirect measure of IQ), and since controlling for neurocognitive functioning did not affect the results, we suggest that the current findings could not be completely explained by potential IQ difference between the two groups.

Notwithstanding these limitations, the current study is one of the very few studies specifically examining the prevalence rates of neurological soft signs in healthy aging people, and Chinese in particular. The observed prevalence of neurological soft signs adds further evidence to the geriatric literature on neurological impairments in cognitively intact people, and may provide a base-rate for identifying a potential clinical cut-off in the near future. The moderate associations of the presence of neurological soft signs in the parent-offspring family units suggest a robust heritability [41]. This study suggests that neurological soft signs may represent a potential neurological marker for the early detection of pathological aging diseases such as mild cognitive impairment and Alzheimer's disease. Future studies using a population-based design could be important to cross-validate our findings.

## List of abbreviations

BLK: Blink; CFI: Comparative Fit Index; CNI: Cambridge Neurological Inventory; COMT: catechol-O-methytransferase; GFI: Goodness of Fit Index; IQ: Intellectual Quotient; LISREL: A software for computing the structural equation modeling; L: left; LN span: Letter-Number Span; MMSE: Mini Mental State Examination; NFI: Normed Fit Index; NNFI: Non-Normed Fit Index; R: Right; RMSEA: Root Mean Square Error of Approximation; SEM: structure equation model

## Competing interests

The authors declare that they have no competing interests.

## Authors' contributions

RCKC conceived the idea, designed the study, and wrote the first draft of the paper. XT collected and analyzed the data. HJL, QZ, HHL, YW, CY, XYC, YNW, YFS collected the data and assisted data analysis. PD participated in writing up the paper. All authors read and approved the final manuscript.
